# Old Age

**Published:** 1904-05-14

**Authors:** 


### OLD AGE.

There must be few amongst us philosophical enough to regard the approach of old age with indifference, and it is perhaps the unpleasant nature of the theme that is responsible for our knowing so little about the pathology of senile degenerations. Whatever the explanation may be it is true that text-books of medicine and pathology alike ignore the whole question, or else fail to differentiate between lesions attributable to diminished vitality and those directly due to the circulation in the blood of poisons, whether alcohol, or the toxins of syphilis, or substances produced by bacterial activity taking place in the bowel. A paper on the subject by Dr. Guthrie Rankin[1] is, therefore, as welcome as it is full of interest. He points out that the arterial system leads the van in the development of the body, and also first succumbs to the influence of decay. In early life the body grows because the calibre of the arteries is great in comparison with the size of the heart and permits of access of a large amount of nutritive plasma to the tissues. There is then a rapid pulse-rate and a low blood-pressure. But as growth proceeds the arteries do not increase in amplitude in proportion either to the greater strength of the heart or to the increasing volume of the tissues. The result is a slowing of the pulse-rate and a rise in the blood-pressure as youth merges into adult life. In course of time the arterial walls undergo a gradual change of structure whereby their original elasticity becomes lessened. In consequence they yield more slowly to the blood-waves and do not recover completely; their lumen, therefore, becomes increased. This causes a lowering of the blood-pressure and favours the shrinkage of many capillaries for which there is no longer an active need, on account of the cessation of growth. The capillary narrowing compensates the permanent dilatation of the inelastic arteries, and the blood-pressure is restored to its normal level. Later on, when defective metabolism has led to a more extensive destruction of capillaries, the tension becomes abnormally high, and this marks the first stage of senile degeneration. Decay is thus the necessary, normal, and final stage of development.

The earliest sign which suggests dangers to come is continuous high blood-pressure. The radial artery is felt to be hard and resistant, and can be rolled like a cord under the finger. At the same time there is a muffled prolongation of the first heart sound, and an accentuation of the aortic second sound, and, where high blood-pressure has existed for some time, there will be hypertrophy of the left ventricle. The degenerative process in the arteries is followed by symptoms of disease in the heart, brain, kidneys or lungs. The cerebral symptoms are many and various, such as loss of memory, headache, attacks of giddiness and so forth. Renal symptoms correspond with those usually regarded as due to chronic interstitial nephritis. In the lungs, bronchitis and emphysema, with secondary failure of the right heart, are liable to occur. The early recognition of the condition is of paramount importance, because by suitably regulating the diet and general routine of daily life much may be done to delay the degenerative process. The chief fact to bear in mind is that the tissue-changes which are customarily associated with senility are probably always preceded by abnormal conditions of the arteries.

[1] Practitioner, May.

